# Identifying women at risk for delayed presentation of breast cancer: a cross-sectional study in Estonia

**DOI:** 10.1186/1471-2458-13-947

**Published:** 2013-10-09

**Authors:** Kaire Innos, Peeter Padrik, Vahur Valvere, Evelyn Eelma, Riina Kütner, Jaak Lehtsaar, Mare Tekkel

**Affiliations:** 1 Department of Epidemiology and Biostatistics, National Institute for Health Development, Tallinn, Estonia; 2 Clinic of Hematology and Oncology, Tartu University Hospital, Tartu, Estonia; 3 Clinic of Hematology and Oncology, University of Tartu, Tartu, Estonia; 4 Clinic of Hematology and Oncology, North Estonia Medical Centre, Tallinn, Estonia; 5 Competence Centre for Cancer Research, Tallinn, Estonia; 6 Clinic of Surgery, North Estonia Medical Centre, Tallinn, Estonia

**Keywords:** Breast cancer, Delayed presentation, Primary care, Women’s health, Smoking, Mammography

## Abstract

**Background:**

Survival from breast cancer remains lower in Estonia than in most other European countries. More advanced stage and larger tumors that have impact on survival may be a result of delay in seeking help for breast cancer symptoms. The aim of this study was to identify determinants of delayed presentation among breast cancer patients in Estonia.

**Methods:**

The study population included women with primary breast cancer diagnosed in Estonia in 2008–2010. All data were collected using structured personal interviews carried out by trained nurses in the hospital setting. Only patients with self-discovered symptoms were included in this analysis. Patient delay was measured as time elapsing from symptom self-discovery to first medical consultation. The effect of different factors on the likelihood of prolonged delay (>90 days) was evaluated using logistic regression.

**Results:**

Among 703 eligible patients, median patient delay was 16 days (interquartile range 5–54 days). Seventeen percent of the patients had their first medical consultation more than three months after self-detection of symptoms. In multivariate analysis, the risk of prolonged delay was significantly associated with age 65 years and over (OR 2.27, 95% CI 1.23–4.20), current smoking (OR 2.09, 95% CI 1.21–3.61), symptoms other than painless breast lump or breast pain (OR 1.84, 95% CI 1.08–3.16), no history of mammograms (OR 1.83, 95% CI 1.13–2.95), having received no information on breast cancer during past year (OR 1.77, 95% CI 1.05–2.99), and previous benign breast problems (OR 1.65, 95% CI 1.01–2.67). Non-significant risk increase was seen with lower education.

**Conclusions:**

This study provides evidence that factors associated with delayed presentation of breast cancer in Eastern Europe are similar to those observed in Western countries. The results suggest that educational messages to general population should aim at increasing awareness of “non-lump” symptoms of breast cancer and encouraging women of all ages to present in a timely manner. Women at risk for delayed presentation such as smokers and women with no history of mammograms could be identified in the primary care setting.

## Background

Breast cancer (BC) is the leading cause of cancer deaths among women in Estonia [1]. Survival from BC, although improving, is still much lower in Estonia than in most other European countries: the estimated 5-year relative survival rate of women diagnosed with BC in Estonia was 74% in 2005–2009 [2], while in most other European countries the respective estimate exceeded 80% already in 2004 [3]. Diagnosing BC at localized stage is less frequent in Estonia (44% in 2005–2007) compared to other countries [2]. The results of a recent study where large regional differences were observed in stage distribution at diagnosis suggested the effect of unemployment, education, and health care access [4].

One possible reason for more advanced stage and larger tumors is delay in seeking help for BC symptoms. The time interval between the patient first noticing a symptom and first consulting a doctor is often referred to as patient delay. Studies in European countries have shown that between 17–20% of patients with BC symptoms delay their presentation for three months or more [5-7]. A recent review showed that longer delay by patients with BC symptoms was associated with sociodemographic factors such as older age, lower level of education and ethnic origin, symptom type and non-recognition of symptom seriousness, while social support/advice had an opposite effect [8]. Other studies have also reported associations with the history of benign breast problems and health care utilization [5,9,10].

This study was undertaken to identify factors that may predict delay by patients with BC symptoms in Estonia, one of the new member states of the European Union that regained its independence after the collapse of the Soviet Union in 1991. We are not aware of any such studies in the countries of economic and health care transition in Eastern Europe.

## Methods

The study population included women diagnosed with morphologically verified primary in situ or invasive BC in Estonia in 2008–2010. There are two specialized oncology centers in Estonia and BC patients are almost exclusively managed at these two hospitals. The patients were recruited from May 2008 to December 2010 at the North Estonia Medical Centre and from December 2008 to December 2010 at Tartu University Hospital. All data for this study were collected using personal interviews carried out by trained nurses in the hospital setting after obtaining written informed consent from each patient. In most cases, the interview was conducted immediately before or after first surgery. The interview included questions on sociodemographic and socioeconomic factors, health behavior, medical history, past mammograms, family history, social support (measured as the number of close persons one can confide in), etc. The patients were also asked to specify whether the first indication of the disease was self-discovered symptoms (breast lump, breast pain, discharge, change of breast shape or size, etc.); abnormal screening/prophylactic mammogram; or a finding by a physician.

In addition, the patients were asked to report their pathway from the first indication of the disease to final diagnosis, including the dates for self-discovery of initial symptoms and the first medical consultation. The current health care system in Estonia is based on national health insurance that relies on the principle of solidarity [11]. Insured persons are those for whom it is required to pay social tax (compulsory for all employers), who pay the social tax for themselves, or who have an equal status with an insured person (e.g. pregnant women, children, retired persons, etc.). Each insured person is listed with a family physician, which function as gatekeepers to the rest of the health-care system. Only a few specialists, including a gynecologist can be visited without a referral. Mammograms are free with a referral or within the screening program. Currently, screening mammography is offered every two years to women age 50–62 years who are covered by national health insurance [4]. The proportion of women covered by national health insurance was 95.5% in 2010 [12].

Cases not discovered by the patients (or their partners) were excluded from this analysis. Initial symptoms were classified into three groups based on the predominant symptom: 1) painless breast lump (with or without other symptoms); 2) breast pain (with or without lump or other symptoms); 3) other symptoms.

The first visit after discovering symptoms was classified into five categories: family physician, gynecologist, mammography, cancer specialist (oncologist or breast surgeon), or other. Those women who visited a cancer specialist as their first appointment were being monitored due to previous cancers of other sites or benign breast problems requiring follow-up.

Prolonged patient delay was defined as an interval of >90 days between the dates for self-discovery of initial symptom and the first medical consultation. This cut-off point was chosen based on previous publications showing that women experiencing delay of three months or more had significantly lower survival [13]. To evaluate the effect of different factors on the likelihood of prolonged delay, logistic regression was used to estimate odds ratios (OR) with 95% confidence intervals (CI). The data were checked for interactions and collinearity. All analyses were done with STATA 12.0 (Stata, College Station, TX, USA).

Variables selected for multivariate regression were those of a priori interest, possible confounders and other factors significantly associated with prolonged delay in univariate analyses. For some variables, data was missing for a few subjects; they were excluded from the logistic regression analyses.

The study was approved by the Tallinn Medical Research Ethics Committee.

## Results

Among the 1195 identified cases, 115 women refused and 54 were not included for other reasons such as mental condition, the refusal of treating physician, etc. The total number of participants was thus 1026 (86%). After the exclusion of 166 cases detected by screening mammography, 61 prophylactic mammography cases, 68 physician-detected cases, 12 cases whose cancer diagnosis was not confirmed morphologically after surgery, 6 cases with known previous BC, and 10 cases with missing data on patient delay, 703 self-detected cases were included in this analysis.

Median patient delay was 16 days (Table 1). Thirty three percent of the patients had a medical appointment within one week of initial symptom discovery, 31% within 8–30 days, and 19% within 31–90 days; 17% experienced delay of >90 days. Mean age at the time of first symptom was 61 years (range 23–92 years).

**Table 1:** Characteristics of women with newly diagnosed primary BC in Estonia, 2008–2010

			**Patient delay (days)**	
	**n**	**%**	**Median**	**Interquartile range**
Total	703	100.0	16	5–54
Age at first symptom (years)				
<50	173	24.6	14	5–36
50–64	199	28.3	15	5–43
≥65	331	47.1	19	5–66
Year of interview				
2008	124	17.6	14	6–38
2009	282	40.1	15	5–54
2010	297	42.3	19	5–66
Nationality				
Estonian	474	67.4	19	6–60
Non-Estonian	229	32.6	11	5–36
Education				
University	172	24.5	11	5–42
Secondary, vocational	399	56.8	16	5–54
Primary, basic	132	18.8	27	8–80
Marital status				
Married/cohabiting	379	53.9	15	5–51
No partner	324	46.1	17	6–58
Active employment				
Yes	306	43.5	14	5–40
No	397	56.5	19	5–61
Self-reported financial status of household				
Very good or good	221	31.4	14	5–47
Satisfactory	393	55.9	18	5–58
Poor or very poor	89	12.7	17	7–34
Smoking status				
Non-smoker	495	70.4	16	5–54
Ex-smoker	91	12.9	11	5–48
Current smoker	117	16.6	19	7–71
Body mass index				
<25	256	36.4	15	5–59
25–29	230	32.7	16	5–48
≥30	213	30.3	16	5–56
Unknown	4	0.6		
No of close persons to confide in				
More than two	385	54.7	18	6–66
One or two	279	39.7	13	5–43
None	36	5.1	12	3–23
Unknown	3	0.4		

The most frequent initial symptom was a painless lump in the breast (Table 2). Over half of the women visited their family doctor first. Overall, 55% of women reported no mammograms before the diagnostic investigations for current disease; the respective proportions were 71% among women age <50 years, 23% among women age 50–64 years and 66% among women age ≥65 years.

**Table 2:** Breast-related characteristics of women with newly diagnosed primary BC in Estonia, 2008–2010

			**Patient delay (days)**	
	**n**	**%**	**Median**	**Interquartile range**
Initial symptom				
Painless breast lump	538	76.5	15	5–52
Breast pain	64	9.1	10	5–35
Other symptoms	101	14.4	22	8–90
First consultation				
Family doctor	411	58.6	13	5–54
Gynecologist	140	19.9	17	6–54
Mammography	59	8.4	12	6–31
Cancer specialist	40	5.7	23	13–56
Other	52	7.4	30	10–94
Previous benign breast problems				
No	519	73.8	15	5–54
Yes	178	25.3	16	5–62
Unknown	6	0.9		
Family history of BC in first degree relatives				5–54
No	619	88.1	16	5–55
Yes	67	9.5	16	5–53
Unknown	17	2.4		
Prior mammograms				
Yes	313	44.5	14	5–37
No	386	54.9	18	5–63
Unknown	4	0.6		
Information on BC during 12 months before initial symptoms				
From at least two sources	352	50.1	13	5–43
From one source	197	28.0	17	5–49
None	150	21.3	28	8–123
Unknown	4	0.6		

In univariate analysis, variables significantly associated with prolonged delay were age, education, the number of close persons, initial symptoms, prior mammograms and information on BC during past 12 months (Table 3). In multivariate logistic regression, the strongest predictors of prolonged patient delay were older age, current smoking, symptoms other than painless breast lump or breast pain, no history of mammograms, no information on BC during past 12 months, and previous benign breast problems. There was a trend towards increased risk with decreasing level of education.

**Table 3:** Risk of patient delay >90 days among women with primary BC in Estonia, 2008–2010

	**No of patients (row%)**^**a**^			
	**No delay (n = 567)**	**Delay >90 days (n = 119)**	**Crude OR (95% CI)**	**Multivariate OR**^**b **^**(95% CI)**
Age at first symptom (years)				
<50	147 (87.0)	22 (13.0)	1.00	1.00
50–64	160 (83.8)	31 (16.2)	1.29 (0.72–2.34)	1.94 (1.00–3.75)
≥65	260 (79.7)	66 (20.3)	1.70 (1.01–2.86)	2.27 (1.23–4.20)
Year of interview				
2008	100 (85.5)	17 (14.5)	1.00	1.00
2009	233 (84.1)	44 (15.9)	1.11 (0.61–2.04)	1.10 (0.58–2.07)
2010	234 (80.1)	58 (19.9)	1.46 (0.81–2.63)	1.48 (0.79–2.77)
Education				
University	145 (87.9)	20 (12.1)	1.00	1.00
Secondary, vocational	322 (82.3)	69 (17.7)	1.55 (0.91–2.65)	1.45 (0.83–2.54)
Primary, basic	100 (76.9)	30 (23.1)	2.17 (1.17–4.05)	1.57 (0.78–3.13)
Smoking status				
Non-smokers, ex-smokers	479 (83.9)	92 (16.1)	1.00	1.00
Current smokers	88 (76.5)	27 (23.5)	1.60 (0.98–2.60)	2.09 (1.21–3.61)
Number of close persons to confide in				
More than two	304 (80.0)	76 (20.0)	1.00	1.00
One or two	229 (84.5)	42 (15.5)	0.73 (0.48–1.11)	0.68 (0.44–1.04)
None	34 (97.1)	1 (2.9)	0.12 (0.02–0.87)	0.09 (0.01–0.67)
Initial symptom				
Painless breast lump	437 (83.9)	84 (16.1)	1.00	1.00
Breast pain	54 (84.4)	10 (15.6)	0.96 (0.47–1.97)	0.94 (0.45–1.98)
Other symptoms	76 (75.2)	25 (24.8)	1.71 (1.03–2.85)	1.84 (1.08–3.16)
Previous benign breast problems				
No	426 (83.5)	84 (16.5)	1.00	1.00
Yes	141 (80.1)	35 (19.9)	1.26 (0.81–1.95)	1.65 (1.01–2.67)
Prior mammograms				
Yes	267 (86.4)	42 (13.6)	1.00	1.00
No	300 (79.6)	77 (20.4)	1.63 (1.08–2.46)	1.83 (1.13–2.95)
Information on BC during 12 months before initial symptoms				
From at least two sources	296 (86.3)	47 (13.7)	1.00	1.00
From one source	161 (83.0)	33 (17.0)	1.29 (0.79–2.10)	1.22 (0.73–2.03)
None	110 (73.8)	39 (26.2)	2.23 (1.38–3.60)	1.77 (1.05–2.99)

^a^patients with missing data excluded.

^b^odds ratio simultaneously adjusted for all variables in the table.

## Discussion

In this study among 703 self-detected BC cases in Estonia, median time between symptom self-discovery and first medical consultation was 16 days. This is in agreement with the findings of other studies in Europe [5,14]. Delay over three months was seen in 17% of the patients, which is within the range observed in previous European studies [5-7]. In a US study, 16% of patients reported similar delay [9], while the same percentage ranged from 25–43% in studies conducted in other parts of the world [15-18]. When comparing these measures across studies, however, it should be born in mind that the study populations have differed slightly from study to study in terms of age restriction, recruitment strategies, etc. (for instance, the Italian study included only surgically treated patients [7] and the US study restricted the age of participants to 30 to 79 [9]).

There was a significant trend in our data towards increased risk of delay with older age. Similar associations have been found in previous studies of BC [5,9,19], as well as of other cancers [20]. Our results may partly explain why older women in Estonia were significantly more likely to be diagnosed with non-localized BC compared with younger women: the proportion of both distant as well as locally advanced (T4) cases was significantly higher among women age 70 years and over compared to younger women in a recent analysis [2]. It has been shown that older women tend to underestimate their risk of developing BC and their knowledge on symptoms other that breast lump is poor [21]. BC related information currently visible for the public in Estonia is mainly addressed to women of screening age (50–62 years) and may create a false perception in older women that this issue does not concern them, thus making them more susceptible to delay.

Our finding that non-lump presenting symptoms increased the risk of delayed presentation is consistent with conclusions from a previous review of evidence [19] as well as with qualitative studies [10,22]. A review showed that the risk of delayed presentation can be increased in patients with many common cancers if a symptom is atypical or vague in nature [8]. In a US study, women holding more misconceptions about breast lumps were more likely to delay presentation [9]. The recognition of symptom seriousness is likely to be related to the patient’s knowledge and awareness of BC. In our study, women who said that they had received no information on BC during one year before first noticing their symptoms had significantly increased risk of prolonged delay compared with those who had received information from at least two sources. Overall, 20% of women reported that they had received no information on BC during past 12 months, clearly indicating the need for making these messages more visible, and reaching all women.

Previous benign breast problems were significantly associated with prolonged delay in our study. The history of benign mastopathy was also found to be a determinant of long patient delay in a study in Germany [5]. A possible explanation for this was suggested by a qualitative study, where the decision not to seek care sooner was influenced by a previous experience of having a breast complaint and being assured that it was benign or a “false alarm” [10].

Most previous studies of patient delay among BC patients have not examined the effect of smoking; however, female smokers experienced longer delays in a Danish study of all cancer patients [23]. In our study, current smokers were two times more likely to present with prolonged delay compared with non-smokers or ex-smokers. Smoking may reflect women’s overall attitudes towards health promoting behavior. Current smoking was shown to be significantly associated with the lack of recent mammograms or Pap smears according to data from the Estonian Health Behaviour Study [24]. No history of mammograms prior to this disease episode was another predictor of prolonged delay in our study that may be associated with overall health behavior as well as health care access and/or utilization. Among US patients, lower health care utilization was significantly associated with prolonged delay [9] while in Germany, non-attendance of general health check-ups predicted prolonged patient delay [5]. Among the participants of this study, mammography use was particularly low among women age 65 and over – 66% of them had never had a mammogram. While screening is currently available only to women age 50–62 years in Estonia, the referral of older women to prophylactic mammography should be more widely used by gynecologists and/or family physicians.

We did not find any associations of delayed presentation with socioeconomic factors except for a non-significant trend with lower education. Consistently with earlier studies, we found no association with marital status [8,19]. It was shown previously that in female cancer patients, the disclosure of symptoms to someone was a more important factor for reducing patient delay than being in a relationship [25]. Similarly, patients with BC who did not disclose their symptoms within a week to someone close to them were shown to be more likely to delay help seeking [19]. Surprisingly, in our study, patients with no social support experienced a significantly decreased risk of delay. However, this should be interpreted with caution as it may be a spurious association due to small numbers; also, the patients were not asked whether they actually disclosed their symptoms to close persons.

The results of this investigation should be considered in light of potential limitations. First, the study relied on patient recall when recording dates, which may have introduced recall bias. However, reliance on self-report for information regarding discovery of a breast symptom is unavoidable [9]. This bias was minimized by interviewing the patients very soon after diagnosis. Second, patient delay as calculated in this study includes possible waiting times and cannot be entirely attributed to patients. While it should be possible to get an appointment with the family physician within one week [11], it may not always be the case, and waiting times for gynecologists tend to be quite long. However, it is not likely for any waiting time to exceed three months. Also, patients with delay >90 days were as likely to visit a family physician first as those with no prolonged delay, and even less likely to visit a gynecologist (data not shown). Third, stage IV patients were underrepresented in our study (1.3% compared to 7.5% among BC patients registered in the Estonian Cancer Registry in 2005–2007 [2]), probably because they were more likely to refuse or to be unable to participate; this may have caused the underestimation of the overall length of delay. No information was available for non-participants. For several variables, data was missing for around one per cent of subjects or less (except for 3% of data on family history of BC); therefore we do not expect that missing data had any meaningful effect on the results.

## Conclusion

In conclusion, this study provides evidence that the factors influencing delayed presentation of symptomatic BC in the countries of health care transition of Eastern Europe are similar to those observed in Western countries. This knowledge should be taken into account when planning and targeting public health activities. In particular, there is ample evidence that BC presenting with symptoms other than a painless lump increases the risk for delayed presentation. Educational messages to the general population should aim at increasing awareness of “non-lump” symptoms, assisting women in symptom recognition, and encouraging earlier presentation, with special emphasis on reaching older women. As a novel finding, we showed that women who tend to neglect their health such as current smokers are more likely to delay presentation, along with women with no history of mammograms. These at-risk groups could be identified in the primary care setting.

## Competing interests

The authors declare that they have no competing interests.

## Authors’ contributions

KI participated in the design of the study, performed the statistical analysis and drafted the manuscript. PP and VV participated in the coordination of the study and data collection, and helped to draft the manuscript. EE, RK and JL participated in the collection of data. MT participated in the design of the study and statistical analysis, and helped to draft the manuscript. All authors read and approved the final manuscript.

## Pre-publication history

The pre-publication history for this paper can be accessed here:

http://www.biomedcentral.com/1471-2458/13/947/prepub
